# Serum BDNF and cognitive risk in maintenance hemodialysis: a machine learning study

**DOI:** 10.1080/0886022X.2026.2732422

**Published:** 2026-09-20

**Authors:** Qiong Tang, Jiakun Tian, Xinyu Ying, Yanyun Zhang, Yanqiao Huo, Daiyao Liu, Yongping Zhang

**Affiliations:** a The First People’s Hospital of Lianyungang, Lianyungang, Jiangsu, China; b Lianyungang Center for Disease Control and Prevention, Lianyungang, Jiangsu, China

**Keywords:** Maintenance hemodialysis, cognitive impairment, brain-derived neurotrophic factor, machine learning, risk prediction, kidney failure

## Abstract

Cognitive impairment is common in maintenance hemodialysis, but practical risk-assessment tools remain limited. We evaluated the association between baseline serum brain-derived neurotrophic factor (BDNF) and 1-year screening-defined incident cognitive impairment and examined whether BDNF added predictive information beyond routine clinical, laboratory, and baseline cognitive variables. This single-center prospective cohort included 130 maintenance hemodialysis patients with baseline Montreal Cognitive Assessment (MoCA) scores ≥26. The primary outcome was screening-defined incident cognitive impairment, defined as follow-up MoCA <26. Serum BDNF was measured by enzyme-linked immunosorbent assay. Five models were assessed using repeated 5-fold cross-validation; repeated nested cross-validation was added as a sensitivity analysis. During follow-up, 70 patients (53.8%) met the screening-defined outcome. Each 1-SD higher BDNF level was associated with lower odds of the outcome [adjusted OR, 0.52 (95% CI, 0.34–0.80); *p* = 0.003]. In the primary analysis, random forest and extreme gradient boosting (XGBoost) had AUCs of 0.798 and 0.797, respectively. Adding BDNF to the XGBoost base model changed AUC from 0.770 to 0.797 (DeLong *p* = 0.236), PR-AUC from 0.746 to 0.779, and Brier score from 0.190 to 0.181. In nested validation, XGBoost AUC was 0.782 (SD 0.019), while paired Base and Base + BDNF AUCs were 0.761 and 0.782, respectively. Lower BDNF was associated with the screening-defined outcome, but its added predictive value was small and statistically uncertain. Serum BDNF may therefore have value as an adjunctive marker for refining risk estimates rather than as a stand-alone predictor; independent multicenter external validation is required before clinical use.

## KEYPOINTS

1. In maintenance hemodialysis patients with baseline MoCA scores ≥26, lower serum brain-derived neurotrophic factor was associated with a higher 1-year risk of screening-defined incident cognitive impairment.
2. Adding serum brain-derived neurotrophic factor to routine baseline variables produced a small and statistically uncertain improvement in discrimination (area under the curve 0.770 to 0.797; DeLong *p* = 0.236), suggesting a modest adjunctive contribution rather than stand-alone predictive value.
3. Serum brain-derived neurotrophic factor may have value as an adjunctive biomarker for cognitive risk stratification in maintenance hemodialysis, but independent multicenter external validation is needed before clinical use.

## Graphical Abstract

**Figure:**
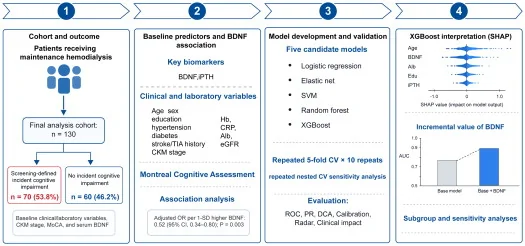

## 1. Introduction

Cognitive impairment is common in chronic kidney disease and has important clinical consequences in maintenance hemodialysis. Even mild impairment can affect medication adherence, dietary management, communication, and shared decision-making. Recent guidance has therefore emphasized the need to recognize cognitive complications in kidney disease [1,2].

The cardiovascular-kidney-metabolic (CKM) framework links kidney disease with cardiovascular and metabolic risk [3]. In this study, all participants already had kidney failure and were receiving maintenance hemodialysis, so the cohort did not span the full CKM spectrum. CKM stage therefore served as a pragmatic descriptor of cardiovascular disease burden, distinguishing stage 3 kidney failure without documented clinical cardiovascular disease from stage 4b kidney failure with documented clinical cardiovascular disease. Accordingly, CKM stage was included as a baseline clinical descriptor rather than as the primary exposure of interest.

Several pathways may contribute to cognitive impairment in hemodialysis, including uremic toxins, anemia, mineral metabolism disorders, inflammation, and vascular disease [4,5]. Dialysis-related stress and broader hemodialysis-related outcome burden may also contribute to cognitive vulnerability [6]. Previous studies in hemodialysis patients have linked cognitive performance with age, cerebrovascular history, physical function, and other clinical factors [7]. These findings support the need for practical risk-stratification tools in dialysis-treated kidney failure.

Current cognitive risk assessment relies mainly on age, comorbidities, routine laboratory tests, cardiovascular history, and screening scores. These variables are useful but may not fully capture neurobiological vulnerability. Brain-derived neurotrophic factor (BDNF), a mediator of neuronal survival and synaptic plasticity, may provide additional information. BDNF has been associated with kidney function and CKD-related biological pathways [8,9]. BDNF has also been linked with cognitive outcomes, but its value in maintenance hemodialysis remains uncertain [10].

Machine learning can integrate clinical and laboratory variables into interpretable risk prediction models [11,12]. Similar prediction approaches have also been explored in maintenance hemodialysis patients [13,14]. We studied maintenance hemodialysis patients with baseline MoCA scores ≥26 to evaluate the association between baseline serum BDNF and 1-year screening-defined incident cognitive impairment and to determine whether BDNF added predictive information beyond baseline clinical variables, CKM stage, laboratory markers, and baseline MoCA. We compared five models, interpreted the XGBoost model, tested the incremental value of BDNF in a paired analysis, and performed sensitivity analyses.

## 2. Materials and methods

### 2.1. Study design and participants

This single-center prospective observational cohort was conducted at the Blood Purification Department of Lianyungang First People’s Hospital. Baseline screening and assessment were completed by January 16, 2025, and 1-year follow-up data were collected through January 16, 2026.

A total of 253 patients receiving maintenance hemodialysis were screened for eligibility and underwent baseline clinical assessment, cognitive screening, and blood sampling. Of these, 103 patients were excluded because of baseline MoCA scores <26, and 19 were excluded because they did not complete the required baseline assessment process owing to incomplete clinical information and/or incomplete questionnaires or assessments. The remaining 131 participants entered prospective follow-up.

Participants entering prospective follow-up had a baseline cognitive assessment and baseline serum BDNF measurement and were scheduled for a 1-year cognitive reassessment. Because the study focused on incident cognitive impairment, patients with baseline cognitive impairment were excluded. Cognitive impairment was defined as a MoCA score <26; the incident-risk cohort therefore included only participants with baseline MoCA scores ≥26.

During the 1-year follow-up, one participant died before the follow-up cognitive assessment. No participants were lost to follow-up or underwent kidney transplantation, and all surviving participants completed the 1-year cognitive assessment. Therefore, 130 participants were included in the final analysis. Of these, 70 met the screening-defined incident cognitive impairment outcome and 60 did not. Participant screening, baseline exclusions, follow-up, and the final analytical cohort are summarized in Figure S4.

### 2.2. Cognitive assessment and outcome definition

Cognitive function was assessed using the Chinese Beijing version of the Montreal Cognitive Assessment (MoCA-BJ). Assessments were administered by study personnel who had received standardized training in MoCA administration and scoring. Standardized instructions and scoring procedures were used at both baseline and the 1-year follow-up. Cognitive assessments were conducted in a quiet clinical environment before the hemodialysis session to minimize the potential influence of dialysis-related fatigue and acute hemodynamic changes.

To account for the influence of education, one point was added to the total MoCA score for participants with ≤12 years of education, with the total score capped at 30 points. We used the conventional cutoff of <26 points to define screening-defined cognitive impairment [15]. Because optimal MoCA thresholds may differ in CKD and kidney failure, this outcome should be interpreted as a screening-based definition rather than a formal neuropsychological diagnosis [16].

The primary outcome was 1-year screening-defined incident cognitive impairment, defined as follow-up MoCA <26 among participants without cognitive impairment at baseline. Baseline MoCA score was retained as a candidate predictor. Follow-up MoCA score and MoCA decline were used only to define or describe outcomes and were not used as predictors.

MoCA decline was calculated as baseline MoCA score minus follow-up MoCA score, with positive values indicating cognitive decline, and was used as a secondary descriptive indicator of cognitive change. Individual baseline-to-follow-up changes in MoCA score are shown in Figure S5.

### 2.3. Serum BDNF measurement and candidate predictors

Serum BDNF was measured at baseline. Peripheral venous blood was collected in the morning before the hemodialysis session and before administration of anticoagulation following an overnight fast. The same blood-sampling protocol was applied to all participants. Serum was separated using standard laboratory procedures. Samples were allowed to clot at room temperature for 30 min and then centrifuged at 2000 × g for 20 min. Baseline blood samples for BDNF measurement from participants included in the analytical cohort were collected over a one-week period. Serum was analyzed immediately or aliquoted and stored at −20 °C for no longer than 7 days before measurement; repeated freeze-thaw cycles were avoided. All baseline serum samples were assayed within this one-week sampling and testing period using a standardized protocol, and serum samples were measured in duplicate. Serum BDNF concentrations were measured using a commercially available human BDNF enzyme-linked immunosorbent assay kit (Human Brain-Derived Neurotrophic Factor ELISA Kit; catalog no. BY-EH112065; Nanjing BYabscience Technology Co., Ltd., Nanjing, China) according to the manufacturer’s instructions. The assay used a double-antibody sandwich ELISA method. Standards and serum samples were added to antibody-precoated microplate wells, followed by horseradish peroxidase-labeled detection antibody. After incubation and washing, substrate solution was added, and the reaction was stopped before optical density was measured at 450 nm. A standard curve was generated using a four-parameter logistic model, and BDNF concentrations were expressed as ng/mL.

According to the manufacturer’s specifications, the assay had a detection range of 1.562–50 ng/mL, a sensitivity of <0.1 ng/mL, an intra-assay coefficient of variation <10%, and an inter-assay coefficient of variation <15%. Because all baseline BDNF measurements were completed before ascertainment of the 1-year cognitive outcomes, laboratory personnel performing the assays were unaware of subsequent cognitive outcome status at the time of measurement. Conducting sample collection and assays within the defined one-week period under a standardized protocol helped limit temporal laboratory variation.

Candidate predictors were selected before modeling based on clinical relevance, data availability, and previous evidence in kidney failure and hemodialysis. Only baseline variables were used. The predictor set included age, sex, education, hypertension, diabetes, stroke or transient ischemic attack history, hemoglobin, C-reactive protein, albumin, eGFR, intact parathyroid hormone, CKM stage, baseline MoCA score, and serum BDNF.

eGFR was retained as a kidney-related laboratory descriptor. In maintenance hemodialysis, however, eGFR should not be interpreted as conventional CKD staging and can only approximate residual kidney function.

CKM stage was assigned according to the American Heart Association framework [3]. Because all participants had kidney failure treated with maintenance hemodialysis, only stage 3 and stage 4b were present. Stage 3 denoted kidney failure without documented clinical cardiovascular disease. Stage 4b denoted kidney failure with documented clinical cardiovascular or cerebrovascular disease recorded in the medical record, including stroke or transient ischemic attack. Stages 0–2 and 4a were not applicable. We used CKM stage as a practical descriptor of cardiovascular/cerebrovascular disease burden within the CKM framework, not as a full CKM phenotype. Missingness is summarized in Table S1.

Follow-up variables, including final MoCA score and MoCA decline, were excluded from model development to reduce information leakage and to ensure that the model used only baseline information.

### 2.4. BDNF association analysis and model development

Baseline characteristics were summarized according to screening-defined incident cognitive impairment status. Continuous variables were reported as mean ± standard deviation or median (interquartile range), depending on distribution, and categorical variables as number and percentage. Between-group comparisons used the t-test or Wilcoxon rank-sum test for continuous variables and the chi-square test or Fisher’s exact test for categorical variables, as appropriate.

Baseline serum BDNF was first compared between patients with and without the screening-defined incident cognitive impairment outcome. It was then categorized into tertiles to describe event rates and MoCA decline across BDNF levels. Restricted cubic spline analysis was used to explore the dose-response relationship between BDNF and the screening-defined cognitive outcome. A joint BDNF-iPTH grouping analysis was used to describe risk patterns across combined biomarker strata. These analyses were descriptive and were not intended to infer causality.

Serum BDNF was standardized using the mean and standard deviation of the overall analytical cohort. Its association with the screening-defined cognitive outcome was estimated using multivariable logistic regression adjusted for age, sex, years of education, diabetes, eGFR, and baseline MoCA score. Results are reported as odds ratios (ORs) with 95% confidence intervals (CIs) per 1-SD higher serum BDNF.

Five candidate models were developed: logistic regression, elastic net, support vector machine, random forest, and XGBoost [17]. These models represented conventional regression, regularized regression, kernel-based learning, bagging, and boosting. Multiple model families were compared to assess the robustness of performance across algorithms rather than to maximize discrimination.

In the primary analysis, model performance was evaluated using repeated 5-fold cross-validation with 10 repeats. Missing-value imputation and scaling, when required, were estimated within resampling, whereas dummy encoding and near-zero-variance filtering were performed before resampling. Hyperparameter selection and performance estimation used the same repeated cross-validation procedure.

To further assess potential optimism arising from preprocessing and model tuning, repeated nested cross-validation was performed as a sensitivity analysis for all five candidate models. The outer loop used stratified 5-fold cross-validation repeated 10 times, while the inner loop used stratified 5-fold cross-validation within each outer-training set for hyperparameter tuning. Missing-value imputation, categorical-level handling, dummy encoding, near-zero-variance filtering, scaling when required, and model tuning were fitted using training-fold data only. Model performance was then evaluated using predictions from the corresponding outer test folds. AUC, PR-AUC, Brier score, calibration intercept, and calibration slope were calculated from the outer-test predictions. For the paired XGBoost comparison, the Base and Base + BDNF models used identical outer and inner resampling folds, with serum BDNF as the only difference in predictor sets. Details of the nested cross-validation analysis are provided in Table S4.

Model performance was assessed using AUC as the primary metric, with PR-AUC, Brier score, calibration intercept and slope, sensitivity, specificity, positive predictive value, and negative predictive value as complementary measures. PR-AUC was included as a complementary measure of positive predictive performance [18]. Calibration was assessed because predicted risks are clinically useful only when probabilities are reliable [19]. Model reporting was guided by TRIPOD+AI recommendations, and risk of bias and applicability were considered in accordance with PROBAST+AI [20,21]. Decision curve analysis was used to describe potential net benefit across threshold probabilities [22]. Details of model tuning and validation are provided in Table S2A.

Model performance was visualized using ROC curves, precision-recall curves, calibration plots, decision curve analysis, radar plots, and a clinical impact curve. The model used for SHAP-based interpretation was selected according to overall performance, including discrimination, prediction error, calibration, probability distribution, decision-curve behavior, and compatibility with model-explanation methods, rather than AUC alone.

### 2.5. SHAP-based model interpretation

SHAP analysis was applied to the selected XGBoost model. SHAP provides an additive feature-attribution framework and estimates each predictor’s contribution to model output [23]. Global importance was summarized using mean absolute SHAP values. Beeswarm plots, dependence plots, and representative explanation plots were used to describe global and individual-level model behavior.

SHAP values were used to characterize model behavior and feature contributions rather than causal effects.

### 2.6. Incremental value and sensitivity analyses

To assess whether BDNF added information beyond routine baseline variables, we compared a Base model with a Base + BDNF model in an XGBoost-based paired incremental analysis. The Base model included demographic variables, clinical history, CKM stage, laboratory indicators, kidney-related variables, and baseline MoCA score. The Base + BDNF model added serum BDNF.

This paired analysis addressed a specific question: how did model performance change when BDNF was added to the same base-feature set? The Base + BDNF model was the all-feature XGBoost model reported in Table 2, and its cross-validated predictions were reused directly. Its performance therefore matched the Table 2 XGBoost result. The Base model used the same repeated cross-validation splits, preprocessing, CKM-stage handling, and selected XGBoost hyperparameters, with serum BDNF removed as the only change.

The incremental value of BDNF was assessed using changes in AUC, PR-AUC, Brier score, calibration, and decision curve performance. AUCs of the paired Base and Base + BDNF models were compared using DeLong’s test for correlated ROC curves. Reclassification was explored descriptively. AUC differences, reclassification measures, and discrimination-slope changes were treated as complementary measures of incremental predictive performance [24,25]. These analyses were intended to characterize incremental predictive information within the development cohort; independent external validation would be required before any clinical implementation [26,27].

Subgroup analyses examined whether the association between BDNF and screening-defined incident cognitive impairment was consistent across clinically relevant strata, including age, diabetes status, eGFR level, and iPTH level. The standardized BDNF variable derived from the overall analytical cohort was used in all subgroup models, so that a 1-SD increase represented the same absolute increase in BDNF across subgroups. The same core adjustment set of age, sex, years of education, diabetes, eGFR, and baseline MoCA score was used, with the corresponding stratification variable omitted when applicable. Median values were used for continuous variables when no established clinical threshold was applied. No formal interaction tests were performed.

Sensitivity analyses assessed whether the BDNF signal depended on baseline cognitive performance or the outcome definition. We first repeated the incremental analysis without baseline MoCA score. We then excluded participants with baseline MoCA = 26 because they were closest to the screening threshold. Finally, we used stricter outcomes requiring follow-up MoCA <26 plus a decline of ≥2 or ≥3 points.

We also examined potential overlap between CKM stage and cardiovascular history. We cross-tabulated CKM stage 4b with stroke/TIA history and calculated phi correlations. We then repeated the Base + BDNF model after removing CKM stage and after removing stroke/TIA history. A prototype model interface is provided in Figure S3 for research demonstration only.

### 2.7. Statistical software

All statistical analyses were performed using reproducible R and Python scripts. Key R analyses were run with R 4.5.3 using xgboost 3.2.1.1, caret 7.0-1, recipes 1.3.2, ranger 0.18.0, pROC 1.19.0.1, PRROC 1.4, dplyr 1.2.1, tibble 3.3.1, readr 2.2.0, and ggplot2 4.0.3. Baseline analyses, model development, model evaluation, nested validation, visualization, and SHAP interpretation were conducted using scripted workflows. The analytic code is available from the corresponding author upon reasonable request. A two-sided *p* value <0.05 was considered statistically significant unless otherwise specified.

## 3. Results

### 3.1. Characteristics of the study population

A total of 253 patients receiving maintenance hemodialysis were screened for eligibility. Of these, 103 were excluded because of baseline MoCA scores <26 and 19 because they did not complete the required baseline assessment process owing to incomplete clinical information and/or incomplete questionnaires or assessments. The remaining 131 participants entered prospective follow-up. During the 1-year follow-up, one participant died before the follow-up cognitive assessment; no participants were lost to follow-up or underwent kidney transplantation. Therefore, 130 participants completed the 1-year cognitive assessment and were included in the final analysis (Figure S4). Of these, 70 patients (53.8%) met the screening-defined incident cognitive impairment outcome and 60 did not. Baseline characteristics by outcome status are shown in Table 1.

**Table 1.** Baseline characteristics according to screening-defined incident cognitive impairment status.

		Incident cognitive impairment status		
Characteristics	Overall	No incident CI	Incident CI	*p* value
**Age, years**	52.00 (42.00, 57.00)	43.00 (38.50, 55.00)	54.00 (47.00, 60.00)	<0.001
**Sex**				0.851
Male	90 (69.2%)	41 (68.3%)	49 (70.0%)	
Female	40 (30.8%)	19 (31.7%)	21 (30.0%)	
**Education, years**	9.00 (8.00, 12.00)	11.00 (9.00, 13.00)	9.00 (8.00, 12.00)	0.002
**History of stroke/TIA**	14 (10.8%)	3 (5.0%)	11 (15.7%)	0.086
**Hypertension**	115 (88.5%)	53 (88.3%)	62 (88.6%)	>0.999
**Diabetes**	30 (23.1%)	12 (20.0%)	18 (25.7%)	0.533
**Hemoglobin, g/L**	110.00 (98.00, 121.00)	113.50 (98.00, 123.00)	109.00 (98.00, 117.00)	0.128
**C-reactive protein, mg/L**	2.92 (1.40, 6.45)	2.48 (0.83, 6.05)	3.25 (1.56, 7.50)	0.212
**Albumin, g/L**	39.60 (36.60, 41.70)	39.75 (38.10, 42.55)	39.35 (35.60, 41.50)	0.093
**eGFR, mL/min/1.73 m²**	6.85 (5.30, 10.90)	6.50 (5.25, 10.10)	7.15 (5.30, 12.80)	0.181
**iPTH, pg/mL**	272.00 (149.00, 493.00)	235.00 (69.60, 526.00)	311.00 (189.00, 467.50)	0.103
**Serum BDNF, ng/mL**	9.53 (7.78, 12.24)	10.31 (8.61, 12.69)	8.75 (7.10, 11.86)	0.004
CKM syndrome stage				0.048
Stage 3	74 (57.8%)	40 (67.8%)	34 (49.3%)	
Stage 4b	54 (42.2%)	19 (32.2%)	35 (50.7%)	
**Baseline MoCA score**				0.881
26	28 (21.5%)	13 (21.7%)	15 (21.4%)	
27	30 (23.1%)	13 (21.7%)	17 (24.3%)	
28	21 (16.2%)	10 (16.7%)	11 (15.7%)	
29	23 (17.7%)	9 (15.0%)	14 (20.0%)	
30	28 (21.5%)	15 (25.0%)	13 (18.6%)	
**Follow-up MoCA score**	25.00 (21.00, 27.00)	27.00 (26.00, 28.00)	21.00 (19.00, 24.00)	<0.001
**MoCA decline**	3.00 (1.00, 7.00)	1.00 (−0.50, 2.00)	6.00 (4.00, 10.00)	<0.001

Values are presented as median (interquartile range) or number (percentage), as appropriate. Between-group comparisons used the t-test, Wilcoxon rank-sum test, chi-square test, or Fisher’s exact test, as appropriate. Screening-defined incident cognitive impairment was defined as follow-up MoCA <26 among participants with baseline MoCA ≥26. CKM stage was interpreted as a pragmatic cardiovascular/cerebrovascular disease-burden descriptor in this maintenance hemodialysis cohort: stage 3 represented kidney failure without documented clinical cardiovascular disease, and stage 4b represented kidney failure with documented clinical cardiovascular or cerebrovascular disease. CKM stage data were available for 128 participants. BDNF, brain-derived neurotrophic factor; CKM, cardiovascular-kidney-metabolic; MoCA, Montreal Cognitive Assessment; TIA, transient ischemic attack.

Patients who developed incident cognitive impairment were older than those who did not [54.00 (47.00, 60.00) vs. 43.00 (38.50, 55.00) years, *p* < 0.001]. They also had fewer years of education [9.00 (8.00, 12.00) vs. 11.00 (9.00, 13.00) years, *p* = 0.002]. Sex distribution was comparable between groups. Stroke or transient ischemic attack history was more common in the incident cognitive impairment group, although this difference was not statistically significant. Hypertension and diabetes were similarly distributed.

Routine laboratory indicators did not differ significantly between groups. Patients who developed incident cognitive impairment tended to have lower hemoglobin and albumin and higher iPTH, whereas CRP and eGFR were similar. Baseline serum BDNF was also lower in patients who developed incident cognitive impairment (Table 1), and this association is detailed in Section 3.2.

CKM stage distribution differed between groups (*p* = 0.048). CKM stage 4b was more frequent among patients who developed incident cognitive impairment [35 (50.7%) vs. 19 (32.2%)]. Among the 54 participants with CKM stage 4b, 13 (24.1%) had stroke/TIA. CKM stage 4b showed a modest phi correlation with stroke/TIA history (phi = 0.360) and strong agreement with recorded cardiovascular disease (agreement, 92.2%; phi = 0.847). This between-group comparison was unadjusted and was not intended to estimate an independent CKM effect.

Baseline MoCA scores were similar between groups, as expected because all included patients were free of cognitive impairment at baseline. At 1 year, the incident cognitive impairment group had lower follow-up MoCA scores [21.00 (19.00, 24.00) vs. 27.00 (26.00, 28.00), *p* < 0.001] and greater MoCA decline [6.00 (4.00, 10.00) vs. 1.00 (−0.50, 2.00), *p* < 0.001]. Individual baseline-to-follow-up MoCA changes are shown in Figure S5.

### 3.2. Serum BDNF distribution and association with the screening-defined cognitive outcome

**Figure 1.**
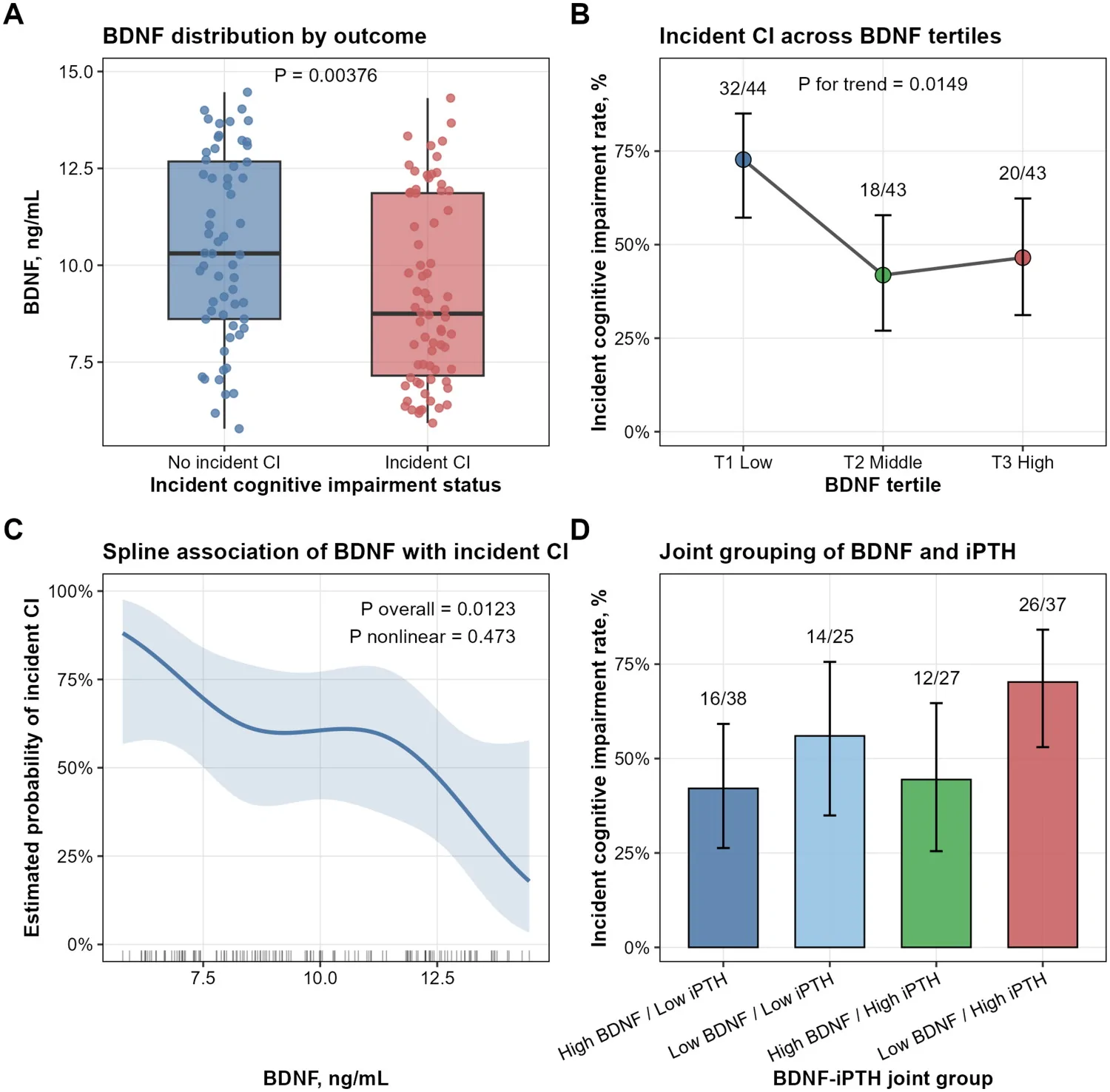
Serum BDNF association analysis. Association analyses of baseline serum BDNF with 1-year screening-defined incident cognitive impairment. Panel A shows the distribution of serum BDNF by outcome status. Panel B shows incident cognitive impairment across BDNF tertiles. Panel C shows the restricted cubic spline association between BDNF and estimated probability of incident cognitive impairment. Panel D shows the joint BDNF-iPTH grouping analysis. Lower BDNF was associated with higher risk, although the tertile pattern was not strictly stepwise. The BDNF-iPTH analysis was exploratory.

Baseline serum BDNF differed between groups (Figure 1A). Patients who developed incident cognitive impairment had lower BDNF levels than those who did not (*p* = 0.004).

When BDNF was categorized into tertiles, the event rate was highest in the lowest tertile (Figure 1B). Event counts were 32/44, 18/43, and 20/43 in the lowest, middle, and highest tertiles, respectively, with a significant trend across tertiles (*p* for trend = 0.015). The decrease was not strictly stepwise from the middle to the highest tertile, but the overall pattern indicated higher risk at lower BDNF levels.

Restricted cubic spline analysis showed that the estimated probability of incident cognitive impairment generally decreased as BDNF increased (Figure 1C). The overall association was statistically significant (*p* overall = 0.012), whereas the test for nonlinearity was not (*p* for nonlinearity = 0.473).

In the joint BDNF-iPTH grouping analysis, patients with low BDNF and high iPTH showed the highest event rate (Figure 1D). This descriptive finding suggests that BDNF may be informative when considered alongside mineral-metabolism indicators, although no formal interaction was tested.

### 3.3. Model performance

**Figure 2.**
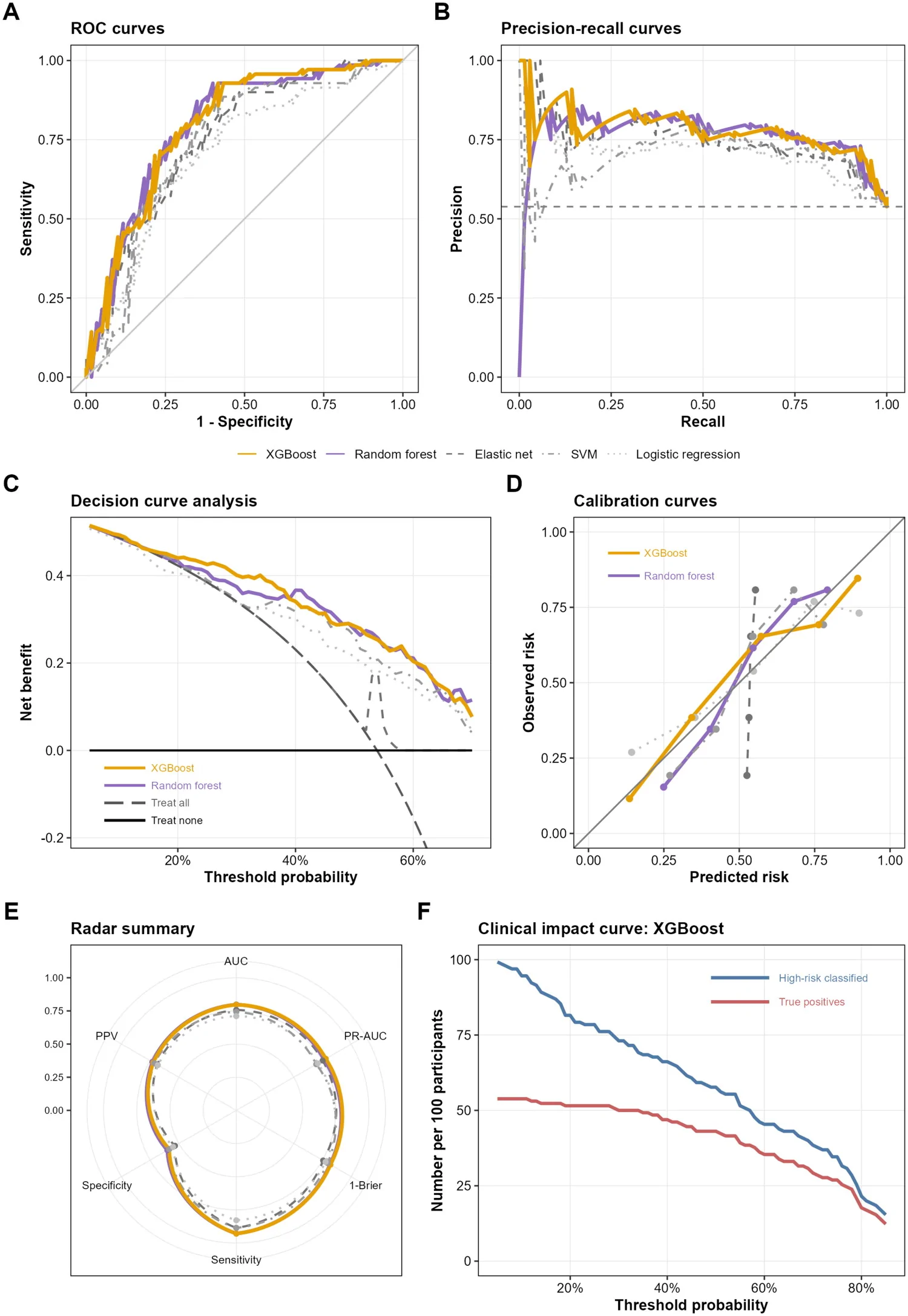
Model performance comparison. Performance of the five candidate models was evaluated using repeated 5-fold cross-validation with 10 repeats. Panels show ROC curves, precision-recall curves, decision curve analysis, calibration curves, a radar summary, and the clinical impact curve for XGBoost. Random forest achieved the highest AUC, whereas XGBoost showed a nearly identical AUC, the highest PR-AUC, and the lowest Brier score. Nested cross-validation results are reported in Table S4.

Five candidate models were compared using repeated 5-fold cross-validation (Table 2 and Figure 2). The distribution of cross-validated AUC, PR-AUC, and Brier score across repeated resampling iterations is shown in Figure S1. Random forest and XGBoost had the highest AUCs: 0.798 (95% CI, 0.718–0.877) and 0.797 (95% CI, 0.717–0.876), respectively. Elastic net, SVM, and logistic regression had lower AUCs of 0.757, 0.743, and 0.711.

**Table 2.** Performance of the five candidate prediction models.

Model	AUC (95% CI)	PR-AUC	Brier score	Calibration intercept	Calibration slope	Sensitivity	Specificity	PPV	NPV
Random forest	0.798 (0.718–0.877)	0.764	0.185	−0.033	1.430	0.929	0.600	0.730	0.878
XGBoost	0.797 (0.717–0.876)	0.779	0.181	0.004	0.867	0.929	0.583	0.722	0.875
Elastic net	0.757 (0.673–0.841)	0.752	0.244	−3.766	25.792	0.886	0.533	0.689	0.800
SVM	0.743 (0.653–0.833)	0.696	0.205	−0.023	1.115	0.886	0.583	0.713	0.814
Logistic regression	0.711 (0.620–0.802)	0.709	0.223	0.059	0.512	0.829	0.550	0.682	0.733

Model performance was evaluated using repeated 5-fold cross-validation with 10 repeats. Random forest achieved the highest AUC, whereas XGBoost showed a nearly identical AUC, the highest PR-AUC, and the lowest Brier score. Threshold-dependent metrics were calculated at the selected model-specific threshold. The extreme calibration slope of elastic net should be interpreted cautiously because its predicted probabilities were narrowly distributed around the overall event rate (Table S2B). Nested cross-validation results are reported separately in Table S4. AUC, area under the receiver operating characteristic curve; PR-AUC, area under the precision-recall curve; XGBoost, extreme gradient boosting.

XGBoost also had the highest PR-AUC (0.779) and the lowest Brier score (0.181). Random forest had slightly higher specificity, while both models showed high sensitivity. Elastic net produced a very narrow probability range around the overall event rate (Table S2B), so its extreme calibration slope should be interpreted cautiously.

The plots were consistent with these findings (Figure 2). XGBoost was used for SHAP interpretation because it balanced discrimination, prediction error, probability distribution, and interpretability. These comparisons reflect internal validation only.

Repeated nested cross-validation yielded somewhat more conservative estimates, while random forest and XGBoost remained the two leading models (Table S4). Across 10 outer repeats, mean (SD) AUC was 0.786 (0.014) for random forest and 0.782 (0.019) for XGBoost. In the paired XGBoost analysis using identical resampling folds, mean AUC was 0.761 (0.025) for Base and 0.782 (0.019) for Base + BDNF, corresponding to a mean paired ΔAUC of 0.021 (SD, 0.014). Mean PR-AUC increased from 0.735 to 0.768, and mean Brier score decreased from 0.195 to 0.187.

### 3.4. SHAP-based model interpretation

**Figure 3.**
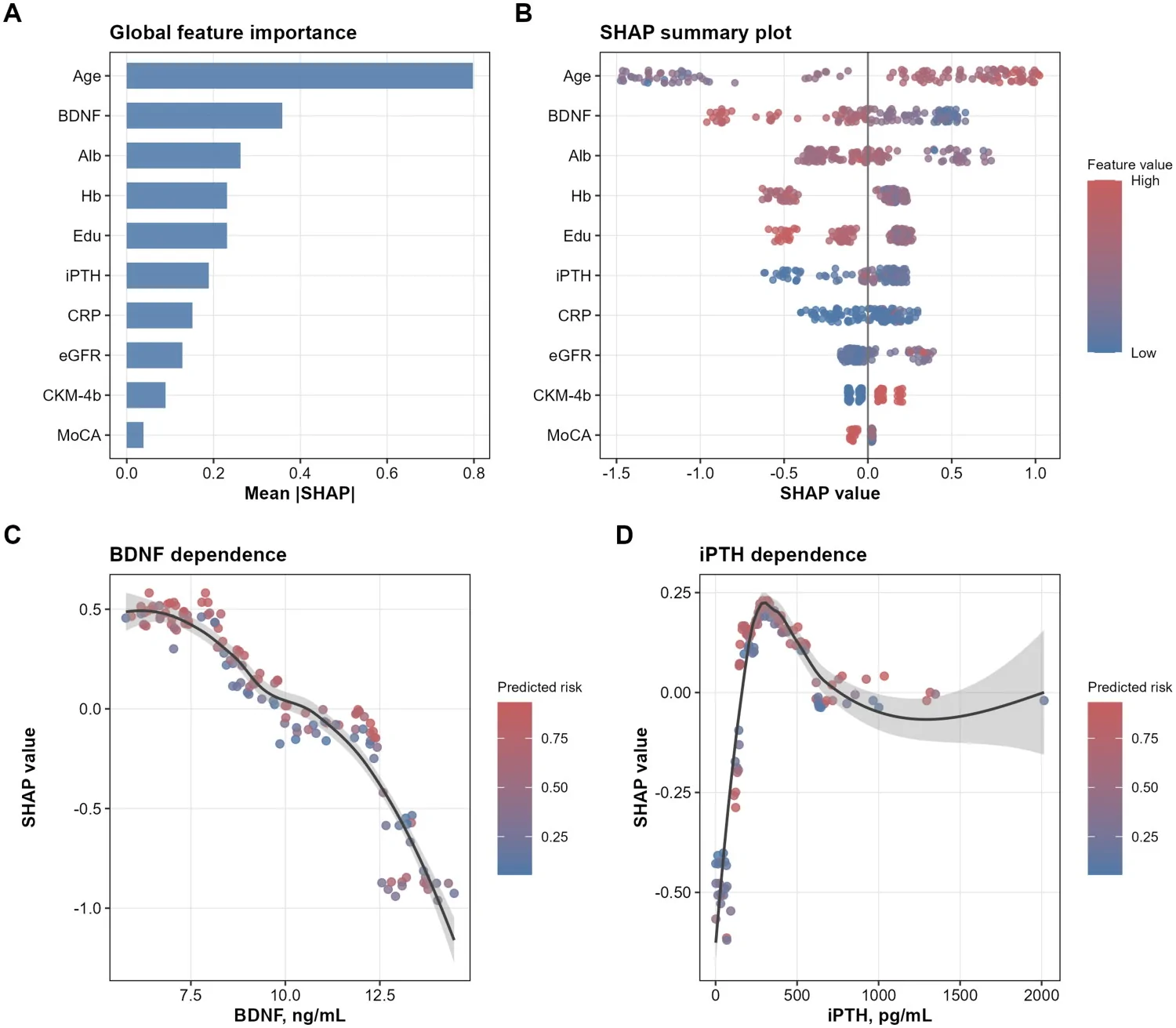
SHAP-based interpretation of the XGBoost model. SHAP-based interpretation of the XGBoost model for predicting 1-year screening-defined incident cognitive impairment. Panel A shows global feature importance ranked by mean absolute SHAP value. Panel B shows the SHAP summary plot. Panel C shows the BDNF dependence plot. Panel D shows the iPTH dependence plot. Age was the leading contributor, followed by BDNF, albumin, hemoglobin, education, and iPTH. CKM stage 4b was interpreted as a pragmatic cardiovascular/cerebrovascular disease-burden descriptor. SHAP values were used to describe model behavior rather than causal effects.

SHAP interpretation of the XGBoost model is shown in Figure 3. Age had the largest mean absolute SHAP value, followed by BDNF, albumin, hemoglobin, education, iPTH, CRP, eGFR, CKM stage 4b, and baseline MoCA score. CKM stage 4b should be interpreted as a pragmatic descriptor of cardiovascular/cerebrovascular disease burden in this cohort rather than as a measure of CKD severity.

Lower BDNF values generally increased predicted risk, whereas higher values reduced model output. This pattern was consistent with the descriptive and spline analyses. The iPTH dependence plot was less linear, suggesting that mineral metabolism markers may contribute differently across their range. SHAP results describe model behavior and should not be interpreted as causal effects.

### 3.5. Incremental value of BDNF

**Figure 4.**
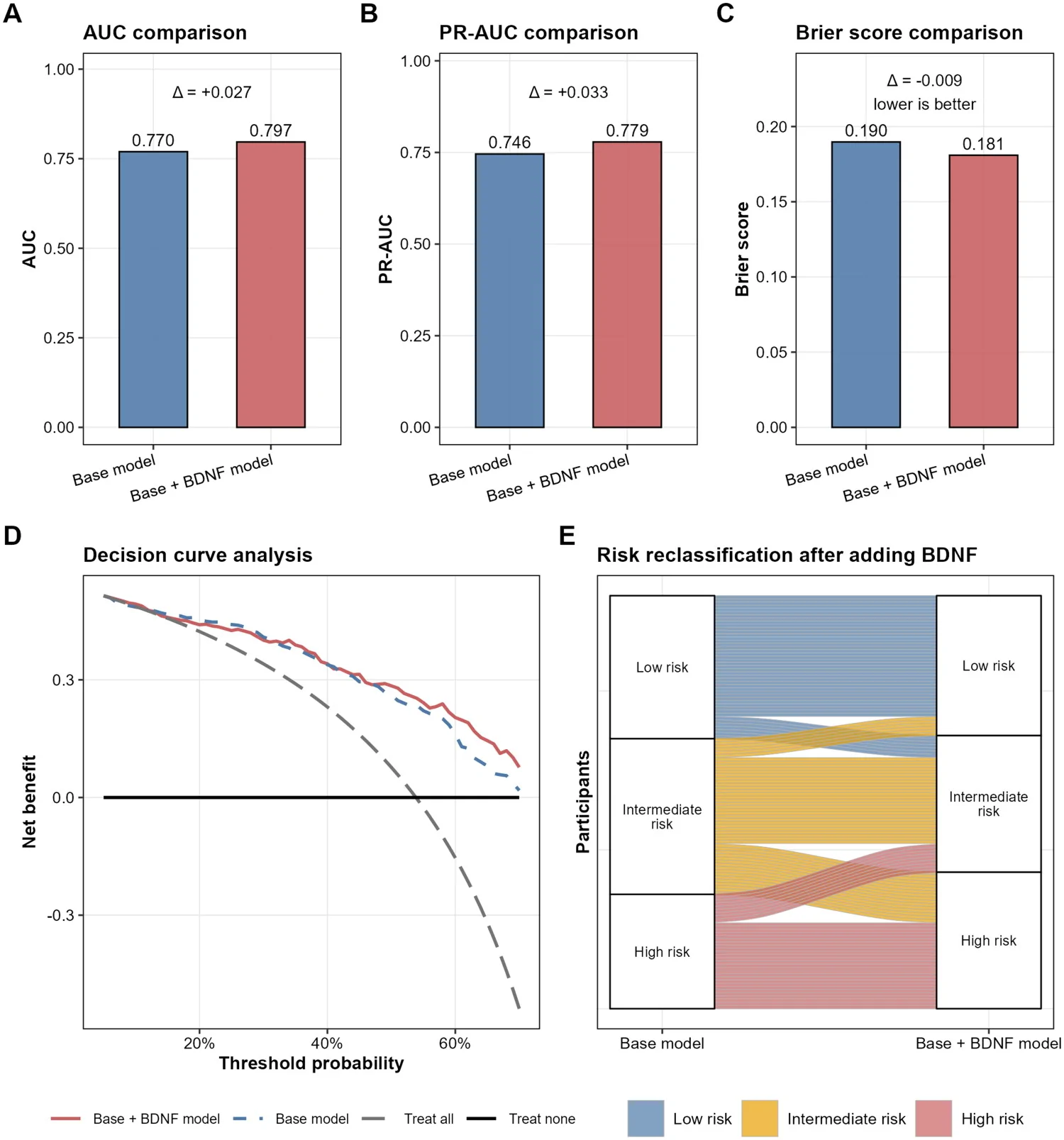
Incremental value of BDNF. Incremental predictive value of adding serum BDNF to the Base model. Panels A-C compare AUC, PR-AUC, and Brier score between the Base model and the Base + BDNF model. Panel D shows decision curve analysis. Panel E shows risk reclassification after adding BDNF. Adding BDNF produced a modest increase in discrimination and PR-AUC with a small reduction in Brier score; the AUC difference was not statistically significant (0.770 vs. 0.797; DeLong *p* = 0.236), and decision-curve and reclassification differences were small.

The incremental value of serum BDNF was evaluated by comparing the Base model with the Base + BDNF model (Table 3 and Figure 4). In the XGBoost-based paired incremental analysis, adding BDNF increased AUC from 0.770 to 0.797 (Figure 4A) and PR-AUC from 0.746 to 0.779 (Figure 4B). The Brier score decreased from 0.190 to 0.181 (Figure 4C). The AUC difference was small and was not statistically significant in a paired DeLong test (*p* = 0.236).

**Table 3.** Incremental predictive value of BDNF.

Model	AUC (95% CI)	PR-AUC	Brier score	Discrimination slope	DeLong *p* for ΔAUC
Base model	0.770 (0.685–0.854)	0.746	0.190	0.270	
Base + BDNF model	0.797 (0.717–0.876)	0.779	0.181	0.294	
Incremental change	0.027	0.033	−0.009	0.024	0.236

The Base + BDNF model corresponded to the all-feature XGBoost model reported in Table 2. The Base model used the same repeated cross-validation splits, preprocessing steps, CKM-stage handling, and selected XGBoost hyperparameters, with serum BDNF removed as the only change. Negative changes in Brier score indicate improved prediction accuracy. The DeLong *p* value was exploratory and was calculated for the paired difference in AUC.

Decision curve analysis showed similar net benefit curves for the two models, with small differences at selected thresholds (Figure 4D). In the reclassification plot, most participants remained in similar risk strata, while some moved between adjacent categories (Figure 4E). Overall, BDNF provided a small but statistically uncertain improvement in predictive performance beyond the Base model.

### 3.6. Subgroup and sensitivity analyses

Subgroup analyses are shown in Figure S2A and Table S3A. Overall, each 1-SD higher serum BDNF level was associated with lower odds of screening-defined incident cognitive impairment [adjusted OR, 0.52 (95% CI, 0.34–0.80), *p* = 0.003]. The inverse direction was generally consistent across age, diabetes, eGFR, and iPTH strata, although some subgroup estimates were imprecise. The association was more evident among patients at or above the median age [adjusted OR, 0.44 (95% CI, 0.25–0.79), *p* = 0.006], patients without diabetes [adjusted OR, 0.53 (95% CI, 0.33–0.87), *p* = 0.012], and patients with higher iPTH [adjusted OR, 0.51 (95% CI, 0.27–0.96), *p* = 0.037].

Sensitivity analyses are summarized in Figure S2 and Tables S3B, S3C, S3D1, and S3D2. In supportive paired Base versus Base + BDNF analyses, adding BDNF increased the AUC from 0.669 to 0.697 in logistic regression and from 0.782 to 0.800 in XGBoost models without baseline MoCA.

Outcome-robustness analyses showed the same direction. After excluding participants with a baseline MoCA of 26, the event rate was nearly unchanged (55/102, 53.9%), the BDNF association persisted [OR per 1-SD higher BDNF, 0.65 (95% CI, 0.43–0.97); *p* = 0.036], and the AUC changed from 0.745 to 0.766 after adding BDNF. Stricter outcomes requiring a follow-up MoCA <26 plus a decline of ≥2 or ≥3 points also preserved the inverse BDNF association (66 and 62 events; OR, 0.63 and 0.65; *p* = 0.011 and 0.019, respectively). Figure S5 shows individual baseline-to-follow-up MoCA score changes, with reference lines at 0, 2, and 3 points. Feature-overlap analyses showed little change after CKM stage or stroke/TIA history was removed. The Base + BDNF AUC was 0.797 in the full model, 0.792 without CKM stage, and 0.796 without stroke/TIA history. Removing either variable changed the AUC by no more than 0.005, showing that model performance was largely unchanged after either variable was removed.

## 4. Discussion

In this single-center prospective cohort of maintenance hemodialysis patients, lower baseline serum BDNF was associated with a higher 1-year risk of screening-defined incident cognitive impairment. BDNF also added a small amount of information to an XGBoost-based prediction model. The gain was modest, but the direction was consistent across several sensitivity analyses. More than half of the cohort developed screening-defined cognitive impairment during follow-up. Because MoCA is a screening instrument, the 53.8% event rate should be understood as a screening-defined cognitive outcome rather than the incidence of clinically diagnosed cognitive impairment. This high event rate did not appear to be driven only by patients starting near the MoCA threshold, because excluding those with a baseline MoCA score of 26 left the event rate almost unchanged and preserved the BDNF association. The same direction was retained when the outcome additionally required MoCA declines of at least 2 or 3 points, with 66 and 62 events, respectively. Together with the individual baseline-to-follow-up changes shown in Figure S5, these findings suggest that the high event rate was not explained solely by minimal crossing of the MoCA threshold. Uremic toxins and CKD-related biological stressors have been proposed as contributors to cognitive dysfunction in kidney disease [28]. Recent hemodialysis cohorts have also reported heterogeneous cognitive-impairment patterns and related clinical or psychological factors [29]. High prevalence of cognitive impairment has been described in maintenance hemodialysis populations [30].

The CKM framework helped describe cardiovascular and cerebrovascular disease burden in this cohort. Because every participant had kidney failure treated with hemodialysis, CKM stages 0–2 and 4a were not represented. Staging therefore separated patients without documented clinical cardiovascular disease (stage 3) from those with documented clinical cardiovascular or cerebrovascular disease (stage 4b), rather than capturing the full adiposity and metabolic dimensions of CKM syndrome. Stage 4b was more common among patients who developed incident cognitive impairment, although this was an unadjusted between-group difference and does not establish an independent CKM association. The observed pattern is biologically plausible given the links among cardiovascular disease, cerebrovascular injury, endothelial dysfunction, inflammation, dialysis-related stress, and cognition. Brain structural changes have also been described in CKD-related cognitive impairment [31]. Clinical marker studies in end-stage kidney disease and hemodialysis have reported associations between cognitive impairment and multiple risk factors [32,33]. In this study, CKM stage should be read as a pragmatic cardiovascular/cerebrovascular disease-burden descriptor, not as a causal exposure.

The model findings are consistent with known risk factors for cognitive impairment in dialysis. Age was the strongest contributor, while albumin and hemoglobin were also influential, in keeping with the roles of nutrition and anemia in hemodialysis outcomes. Screening-based studies have linked MoCA scores with nutritional assessment in ESKD [34]. Cognitive impairment and malnutrition have also been discussed as overlapping problems in hemodialysis [35]. Anemia has been associated with cognitive impairment in maintenance hemodialysis, supporting the relevance of hemoglobin in the model [36]. BDNF ranked among the leading predictors, suggesting that neurotrophic status may carry information not fully captured by routine clinical variables. Decreased BDNF expression has been reported in CKD-related clinical and experimental evidence [37]. Higher serum BDNF has also been associated with lower risk of poststroke cognitive impairment [38]. BDNF-related biomarkers have been examined in children with CKD and in adults with mild cognitive impairment or dementia [39,40]. Plasma BDNF/irisin ratio has also been related to cognitive function in older adults [41]. In this cohort, patients who developed screening-defined incident cognitive impairment had lower BDNF levels, and spline analysis showed an inverse association without clear nonlinearity. These observations support biological plausibility but do not establish causality.

Random forest and XGBoost performed similarly in the primary analysis. XGBoost was chosen for interpretation because it combined good discrimination, a low Brier score, a broad risk distribution, and SHAP compatibility. Repeated nested cross-validation yielded somewhat more conservative estimates, while random forest and XGBoost remained the two leading models, with mean AUCs of 0.786 and 0.782, respectively. The paired nested analysis also retained a small positive change after adding BDNF, with mean AUC increasing from 0.761 to 0.782. These findings support the overall pattern observed in the primary analysis while providing a more stringent estimate of internal performance. Independent external validation remains necessary to assess transportability.

Overall, BDNF appeared to add modest predictive information. The tertile analysis showed the highest risk in the lowest tertile but was not strictly stepwise, arguing against BDNF as a stand-alone screening marker. In the paired incremental analysis, adding BDNF produced small improvements in AUC, PR-AUC, and Brier score; the AUC difference was not statistically significant, and decision-curve differences were also small. The paired nested analysis showed a similar direction, with a mean AUC increase of 0.021 and accompanying improvements in PR-AUC and Brier score. The signal persisted after excluding participants near the baseline MoCA threshold, after applying stricter outcome definitions requiring both a follow-up MoCA <26 and a minimum decline, and after removing CKM stage or stroke/TIA history. These analyses help address concerns about threshold-score regression and cardiovascular-variable overlap. The joint BDNF-iPTH analysis was descriptive, but the highest event rate occurred in patients with low BDNF and high iPTH. Secondary hyperparathyroidism has been discussed in relation to cognitive decline, supporting the biological plausibility of the iPTH-related descriptive pattern [42]. BDNF is therefore better described as an adjunctive biomarker that may refine risk estimates, rather than as a decisive predictor. Its clinical value will require confirmation in larger external cohorts.

This study has several strengths. It focused on incident rather than prevalent cognitive impairment, used only baseline predictors to limit information leakage, and examined BDNF through complementary association, prediction, interpretation, incremental-value, and sensitivity analyses. Model performance was evaluated using discrimination, calibration, Brier score, decision curves, and probability distributions rather than AUC alone. The additional nested cross-validation analysis further separated model tuning from outer-fold performance estimation and provided a more stringent assessment of internal model performance.

Several limitations should be considered. The study was single-center and included only 130 participants. With 70 events and about 14 candidate predictors, the events-per-variable ratio was approximately 5, and overfitting cannot be excluded despite repeated and nested cross-validation. External validation and recalibration are therefore required before clinical use. In addition, 19 screened patients did not complete the required baseline assessment process, and one participant died before the 1-year cognitive assessment; these factors may limit the representativeness of the final analytical cohort. The outcome was based on MoCA screening rather than a full neuropsychological battery. Although the high 1-year event rate may partly reflect threshold effects and test-retest variability, the individual changes shown in Figure S5 and the analyses requiring declines of at least 2 or 3 points indicate that many events were accompanied by more substantial score reductions. Future studies should use repeated, domain-specific cognitive testing. Given the single-center development design, external validation remains an important next step before the model is considered for clinical application.

Other limitations relate to predictor definitions. CKM stage was derived from available records and mainly functioned as a marker of cardiovascular/cerebrovascular disease burden. eGFR is difficult to interpret during maintenance hemodialysis and cannot replace measured residual kidney function or urine output. BDNF was measured only once, and peripheral levels may be affected by biological variability despite standardized blood collection and assay procedures. Accordingly, a single baseline measurement may not fully capture longitudinal changes in neurotrophic status. Dialysis-specific variables, including dialysis vintage, adequacy, access type, interdialytic weight gain, and intradialytic hypotension, were not fully captured. Larger multicenter studies are needed to evaluate reproducibility, calibration, transportability, and clinical usefulness.

## 5. Conclusion

This single-center prospective cohort developed and internally evaluated a prediction approach for 1-year screening-defined incident cognitive impairment in maintenance hemodialysis patients. Lower baseline serum BDNF was associated with higher risk and added a small but statistically uncertain amount of predictive information beyond routine baseline variables, CKM stage, and baseline cognitive performance.

Serum BDNF may help refine cognitive risk stratification as an adjunctive biomarker. However, the incremental improvement was modest and did not reach statistical significance for AUC, CKM stage mainly reflected documented cardiovascular/cerebrovascular disease burden, and validation was internal. These findings support further evaluation of BDNF as an adjunctive biomarker rather than routine clinical implementation at this stage. Multicenter external validation is needed before this approach can be considered for clinical use.

## Supplementary Material

FigureS5.tiff

Figure S3.tiff

FigureS1.tiff

FigureS2.tiff

FigureS4.tiff

Supplement Clean.docx

## Data Availability

The de-identified datasets generated and analyzed during the current study are available from the corresponding author upon reasonable request, subject to approval by the Medical Ethics Committee of the First People’s Hospital of Lianyungang and applicable privacy regulations.

## Clinical trial number

Not applicable. This study was an observational cohort study and was not a clinical trial.
