# Supplementary material for: Serum BDNF and cognitive risk in maintenance hemodialysis: a machine learning study
Source: Ren Fail. 2026 Sep 20;48(1):2732422. doi: 10.1080/0886022X.2026.2732422 (PMC13592005; doi:10.1080/0886022X.2026.2732422)
Supplement: Supplementary file 1 [file IRNF_A_2732422_SM5860.docx]

Table S1. Missingness of study variables in the final analysis cohort.

| **raw_variable** | **display_name_table** | **role** | **unit** | **total_n** | **non_missing_n** | **missing_n** | **missing_rate** | **missing_percent** |
| --- | --- | --- | --- | --- | --- | --- | --- | --- |
| ipth_pg_ml | iPTH, pg/mL | Biomarker | pg/mL | 130 | 127 | 3 | 0.023076923 | 2.31% |
| ckm_stage | CKM syndrome stage | Pragmatic cardiovascular/cerebrovascular disease-burden descriptor |  | 130 | 128 | 2 | 0.015384615 | 1.54% |
| hb_g_l | Hemoglobin, g/L | Laboratory predictor | g/L | 130 | 129 | 1 | 0.007692308 | 0.77% |
| age_years | Age, years | Clinical predictor | years | 130 | 130 | 0 | 0 | 0% |
| albumin_g_l | Albumin, g/L | Laboratory predictor | g/L | 130 | 130 | 0 | 0 | 0% |
| baseline_moca_score2 | Baseline MoCA score | Baseline cognitive assessment | points | 130 | 130 | 0 | 0 | 0% |
| bdnf_ng_ml | Serum BDNF, ng/mL | Experimental biomarker | ng/mL | 130 | 130 | 0 | 0 | 0% |
| crp_mg_l | C-reactive protein, mg/L | Laboratory predictor | mg/L | 130 | 130 | 0 | 0 | 0% |
| cvd_recorded_bin | Recorded cardiovascular disease | Recorded CVD descriptor |  | 130 | 130 | 0 | 0 | 0% |
| diabetes_bin | Diabetes | Clinical predictor |  | 130 | 130 | 0 | 0 | 0% |
| education_years | Education, years | Clinical predictor | years | 130 | 130 | 0 | 0 | 0% |
| egfr_ml_min_1_73m2 | eGFR, mL/min/1.73 m² | Laboratory predictor | mL/min/1.73 m² | 130 | 130 | 0 | 0 | 0% |
| female_bin | Sex | Clinical predictor |  | 130 | 130 | 0 | 0 | 0% |
| final_moca_score | Follow-up MoCA score | Follow-up cognitive assessment | points | 130 | 130 | 0 | 0 | 0% |
| hypertension_bin | Hypertension | Clinical predictor |  | 130 | 130 | 0 | 0 | 0% |
| incident_ci_1y | Incident cognitive impairment | Outcome |  | 130 | 130 | 0 | 0 | 0% |
| moca_decline | MoCA decline | Supplementary outcome | points | 130 | 130 | 0 | 0 | 0% |
| stroke_tia_history_bin | History of stroke/TIA | Clinical predictor |  | 130 | 130 | 0 | 0 | 0% |

Missingness was assessed in the final analysis cohort. Baseline variables were used as candidate predictors, whereas follow-up MoCA score and MoCA decline were used for outcome definition and descriptive analyses only and were not included as candidate predictors in model development. Because all participants were receiving maintenance hemodialysis, CKM stage in this dataset distinguished kidney failure without documented clinical cardiovascular disease (stage 3) from kidney failure with documented clinical cardiovascular or cerebrovascular disease (stage 4b). CKM stage was therefore used as a pragmatic cardiovascular/cerebrovascular disease-burden descriptor within the CKM framework rather than as a full-spectrum CKM phenotype.

Table S2A. Model tuning and validation settings.

| **Model** | **Algorithm** | **R method/package** | **Candidate predictors** | **Preprocessing** | **Validation strategy** | **Hyperparameters evaluated** | **Selected/final settings** | **Role in analysis** |
| --- | --- | --- | --- | --- | --- | --- | --- | --- |
| Logistic regression | Penalization-free multivariable logistic regression | glm | Baseline demographic, clinical, pragmatic CKM-stage descriptor, laboratory, baseline MoCA, iPTH, and serum BDNF variables | Primary: fold-wise median imputation and scaling; dummy encoding and near-zero-variance filtering before repeated CV. | Repeated 5-fold cross-validation with 10 repeats | No hyperparameter tuning | Not applicable | Clinical reference model |
| Elastic net | Elastic-net penalized logistic regression | glmnet | Same candidate predictors as above | Primary: fold-wise median imputation and scaling; dummy encoding and near-zero-variance filtering before repeated CV. | Repeated 5-fold cross-validation with 10 repeats | alpha, lambda | alpha = 0; lambda = 10; the selected penalty corresponded to ridge regression within the elastic-net framework | Regularized linear machine-learning model |
| SVM | Support vector machine classifier | svmRadial | Same candidate predictors as above | Primary: fold-wise median imputation and scaling; dummy encoding and near-zero-variance filtering before repeated CV. | Repeated 5-fold cross-validation with 10 repeats | sigma, C | sigma = 0.0466976728930172; C = 2 | Nonlinear classifier |
| Random forest | Random forest classifier | ranger | Same candidate predictors as above | Primary: fold-wise median imputation; dummy encoding and near-zero-variance filtering before repeated CV. | Repeated 5-fold cross-validation with 10 repeats | mtry, splitrule, min.node.size | mtry = 4; splitrule = gini; min.node.size = 10 | Tree-based ensemble classifier |
| XGBoost | Extreme gradient boosting classifier | xgboost | Same candidate predictors as above | Primary: fold-wise median imputation; dummy encoding and near-zero-variance filtering before repeated CV. | Repeated 5-fold cross-validation with 10 repeats; final full-data model was refitted for SHAP interpretation and supplementary research demonstration | nrounds, max_depth, eta, subsample, colsample_bytree, min_child_weight, gamma | nrounds = 300; max_depth = 3; eta = 0.03; subsample = 0.80; colsample_bytree = 0.80; min_child_weight = 3; gamma = 0; objective = binary:logistic; eval_metric = auc | Selected model for SHAP interpretation based on balanced overall performance |

Summary of candidate algorithms, preprocessing procedures, resampling design, hyperparameters, selected settings, and analytical role. In the primary analysis, missing-value imputation and scaling when required were performed within resampling, whereas dummy encoding and near-zero-variance filtering preceded repeated cross-validation. Hyperparameter selection and performance estimation used the same repeated 5-fold cross-validation with 10 repeats. The full-data XGBoost model was refitted only for SHAP interpretation and the supplementary research demonstration, not for estimating cross-validated performance. A separate nested sensitivity analysis with fold-specific preprocessing, inner-loop tuning, and outer-loop evaluation is reported in Table S4.

Table S2B. Distribution of cross-validated predicted probabilities.

| **Model** | **n** | **Event rate** | **Min** | **P1** | **P5** | **P25** | **Median** | **Mean** | **P75** | **P95** |
| --- | --- | --- | --- | --- | --- | --- | --- | --- | --- | --- |
| XGBoost | 130 | 0.538 | 0.039 | 0.055 | 0.104 | 0.288 | 0.569 | 0.541 | 0.791 | 0.928 |
| Logistic regression | 130 | 0.538 | 0.032 | 0.046 | 0.106 | 0.310 | 0.541 | 0.538 | 0.772 | 0.940 |
| Random forest | 130 | 0.538 | 0.121 | 0.160 | 0.210 | 0.375 | 0.543 | 0.535 | 0.708 | 0.828 |
| SVM | 130 | 0.538 | 0.125 | 0.163 | 0.216 | 0.402 | 0.535 | 0.540 | 0.699 | 0.797 |
| Elastic net | 130 | 0.538 | 0.515 | 0.520 | 0.524 | 0.531 | 0.539 | 0.539 | 0.545 | 0.556 |

Distribution of cross-validated predicted probabilities for the five candidate models. The probability-distribution check was used as a supplementary model diagnostic. XGBoost produced predicted probabilities with a mean value close to the observed event rate while retaining a wide risk range. Elastic net generated narrowly distributed probabilities around the overall event rate, which was consistent with its less favorable Brier score and unstable calibration-slope estimate.

Table S3A. Subgroup analyses of serum BDNF.

| **Subgroup** | **n** | **events** | **Adjusted OR per 1-SD**  **higher BDNF (95% CI)** | **P value** |
| --- | --- | --- | --- | --- |
| Overall | 130 | 70 | 0.52 (0.34-0.80) | 0.003 |
| Age < median | 63 | 26 | 0.59 (0.31-1.12) | 0.104 |
| Age >= median | 67 | 44 | 0.44 (0.25-0.79) | 0.006 |
| No diabetes | 100 | 52 | 0.53 (0.33-0.87) | 0.012 |
| Diabetes | 30 | 18 | 0.44 (0.16-1.21) | 0.112 |
| eGFR < median | 65 | 34 | 0.47 (0.24-0.92) | 0.027 |
| eGFR >= median | 65 | 36 | 0.55 (0.31-0.98) | 0.044 |
| iPTH < median | 63 | 30 | 0.54 (0.29-1.01) | 0.053 |
| iPTH >= median | 64 | 38 | 0.51 (0.27-0.96) | 0.037 |

Odds ratios were estimated using multivariable logistic regression. Serum BDNF was standardized using the mean and standard deviation of the overall analytical cohort, and the same standardized BDNF variable was used across all subgroup analyses. Thus, a 1-SD increase represented the same absolute increase in serum BDNF across subgroups (approximately 2.46 ng/mL). Models were adjusted for age, sex, years of education, diabetes, eGFR, and baseline MoCA score, with the corresponding stratification variable omitted when applicable. No formal interaction tests were performed.

Table S3B. Sensitivity analyses of the incremental value of serum BDNF.

| **Analysis** | **Model** | **AUC (95% CI)** | **PR-AUC** | **Brier score** | **Discrimination slope** | **n** | **events** |
| --- | --- | --- | --- | --- | --- | --- | --- |
| Logistic sensitivity | Logistic Base | 0.669 (0.574-0.763) | 0.672 | 0.24 | 0.15 | 130 | 70 |
| Logistic sensitivity | Logistic Base + BDNF | 0.697 (0.604-0.790) | 0.685 | 0.23 | 0.195 | 130 | 70 |
| Logistic sensitivity | Logistic incremental change | 0.028 | 0.013 | -0.011 | 0.045 | 130 | 70 |
| XGBoost without baseline MoCA | XGBoost no MoCA Base | 0.782 (0.701-0.864) | 0.771 | 0.185 | 0.281 | 130 | 70 |
| XGBoost without baseline MoCA | XGBoost no MoCA Base + BDNF | 0.800 (0.722-0.878) | 0.785 | 0.179 | 0.298 | 130 | 70 |
| XGBoost without baseline MoCA | XGBoost no MoCA incremental change | 0.018 | 0.013 | -0.006 | 0.017 | 130 | 70 |

Sensitivity analyses evaluated the incremental value of serum BDNF in logistic regression and in XGBoost models excluding baseline MoCA score. Negative changes in Brier score indicate improved prediction accuracy. These analyses were considered supportive and exploratory; no formal statistical test or confidence interval was calculated for incremental differences in AUC or PR-AUC.

Table S3C. Outcome-robustness analyses of serum BDNF.

| **Analysis** | **n/events** | **Event rate (%)** | **OR per 1-SD BDNF (95% CI), P** | **AUC: Base -> Base + BDNF** | **Delta AUC (DeLong P)** |
| --- | --- | --- | --- | --- | --- |
| Exclude baseline MoCA = 26 | 102/55 | 53.9 | 0.65 (0.43-0.97), P=0.036 | 0.745 -> 0.766 | 0.021 (0.373) |
| Strict outcome: MoCA <26 + decline >=2 | 130/66 | 50.8 | 0.63 (0.44-0.90), P=0.011 | 0.759 -> 0.777 | 0.018 (0.428) |
| Strict outcome: MoCA <26 + decline >=3 | 130/62 | 47.7 | 0.65 (0.45-0.93), P=0.019 | 0.703 -> 0.729 | 0.026 (0.269) |

Outcome-robustness analyses included exclusion of participants with baseline MoCA = 26 and stricter outcome definitions requiring follow-up MoCA <26 plus a decline of at least 2 or 3 points from baseline. For these analyses, serum BDNF was standardized within each corresponding analysis sample before estimation of the OR. AUC comparisons used the same XGBoost-based paired incremental framework as the primary incremental analysis. DeLong P values were exploratory.

Table S3D1. Overlap between CKM stage 4b and cardiovascular disease variables.

| **Metric** | **Value** |
| --- | --- |
| Participants with available CKM stage | 128 |
| CKM stage 4b | 54 |
| CKM stage 4b with stroke/TIA | 13 |
| CKM stage 4b without stroke/TIA | 41 |
| Proportion of CKM stage 4b with stroke/TIA (%) | 24.1 |
| Phi correlation: CKM stage 4b vs stroke/TIA | 0.360 |
| Recorded CVD | 45 |
| Agreement: CKM stage 4b vs recorded CVD | 0.922 |
| Phi correlation: CKM stage 4b vs recorded CVD | 0.847 |

CKM, cardiovascular-kidney-metabolic; CVD, cardiovascular disease; TIA, transient ischemic attack. Phi correlations were calculated using complete observations.

Table S3D2. Feature-overlap sensitivity models.

| **Model** | **AUC** | **PR-AUC** | **Brier score** |
| --- | --- | --- | --- |
| Full Base + BDNF | 0.797 | 0.779 | 0.181 |
| Minus CKM stage | 0.792 | 0.776 | 0.182 |
| Minus stroke/TIA history | 0.796 | 0.775 | 0.181 |

Feature-overlap sensitivity analyses examined changes in model performance after removing CKM stage or stroke/TIA history. The full model refers to the Base + BDNF model in the XGBoost-based paired incremental framework.

Supplementary Methods. Nested cross-validation sensitivity analysis.

The outer loop used stratified 5-fold cross-validation repeated 10 times; the inner loop used stratified 5-fold cross-validation for tuning within each outer-training set. All preprocessing parameters were learned within the relevant training fold: numeric missing values were median-imputed; missing and novel categorical levels were handled before dummy encoding; near-zero-variance filtering was fold-specific; and normalization was applied for logistic regression, elastic net, and SVM. Logistic regression had no tuning parameters. Elastic net evaluated alpha values 0, 0.5, and 1 with five lambda values from 0.001 to 10. SVM evaluated sigma values 0.01, 0.05, and 0.10 and C values 0.5, 1, 2, and 4. Random forest evaluated mtry values 3, 6, and 9 and minimum node sizes 3, 5, and 10 with 500 trees. XGBoost evaluated 75 or 150 boosting rounds, maximum depths 2 or 3, and learning rates 0.03 or 0.08, with gamma 0, column sampling 0.80, minimum child weight 3, and subsampling 0.80. No threshold was selected. The XGBoost Base and Base + BDNF analyses used identical outer and inner folds.

Table S4. Nested cross-validation sensitivity analysis.

| **Panel / model** | **Predictor set** | **AUC, mean (SD)** | **PR-AUC, mean (SD)** | **Brier, mean (SD)** | **Calibration intercept, mean (SD)** | **Calibration slope, mean (SD)** |
| --- | --- | --- | --- | --- | --- | --- |
| A. Logistic regression | Base + BDNF | 0.696 (0.019) | 0.705 (0.029) | 0.234 (0.010) | -0.006 (0.051) | 0.415 (0.075) |
| A. Elastic net | Base + BDNF | 0.694 (0.035) | 0.698 (0.033) | 0.231 (0.008) | 0.002 (0.023) | 1.581 (0.687) |
| A. Support vector machine | Base + BDNF | 0.715 (0.014) | 0.714 (0.024) | 0.215 (0.006) | 0.127 (0.120) | 0.854 (0.108) |
| A. Random forest | Base + BDNF | 0.786 (0.014) | 0.764 (0.021) | 0.189 (0.005) | 0.049 (0.045) | 1.233 (0.159) |
| A. XGBoost | Base + BDNF | 0.782 (0.019) | 0.768 (0.025) | 0.187 (0.010) | -0.042 (0.063) | 0.899 (0.137) |
| B. XGBoost | Base | 0.761 (0.025) | 0.735 (0.033) | 0.195 (0.011) | -0.018 (0.067) | 0.868 (0.175) |
| B. XGBoost | Base + BDNF | 0.782 (0.019) | 0.768 (0.025) | 0.187 (0.010) | -0.042 (0.063) | 0.899 (0.137) |
| B. Paired change | Base + BDNF minus Base | +0.021 (0.014) | +0.032 (0.028) | -0.008 (0.006) | Not summarized for change | Not summarized for change |

Values are means (SDs) across 10 complete outer 5-fold repeats. Panel A compares five candidate models using the full Base + BDNF predictor set. Panel B presents the paired XGBoost comparison using identical outer and inner folds, with BDNF as the only predictor-set difference. The nested analysis was performed as a sensitivity analysis to provide a more stringent internal-validation estimate. AUC, area under the receiver operating characteristic curve; BDNF, brain-derived neurotrophic factor; PR-AUC, area under the precision-recall curve; SVM, support vector machine.

Figure S1. Repeated cross-validation distribution.

Distribution of cross-validated AUC, PR-AUC, and Brier score across repeated 5-fold cross-validation resamples. This figure provides an overview of the stability and variability of model performance across resampling iterations.

Figure S2. Subgroup and sensitivity analyses.

Panel A shows subgroup analyses of the association between 1-SD higher serum BDNF and screening-defined incident cognitive impairment. Serum BDNF was standardized using the overall analytical cohort, so the same 1-SD scale was applied across subgroups. Panel B compares logistic regression Base and Base + BDNF models. Panel C compares XGBoost Base and Base + BDNF models after excluding baseline MoCA score.

Figure S3. Prototype research demonstration and representative prediction example.

Representative interface generated from the final full-data XGBoost model using the same baseline predictors as the Base + BDNF model. The interface displays a predicted probability and a tertile-based research risk group derived from the development cohort. It is presented as a research demonstration and has not undergone external validation.

Figure S4. Participant flow diagram.

A total of 253 patients receiving maintenance hemodialysis were screened for eligibility. Of these, 103 were excluded because of baseline MoCA scores <26 and 19 because they did not complete the required baseline assessment process owing to incomplete clinical information and/or incomplete questionnaires or assessments. The remaining 131 participants entered prospective follow-up. During the 1-year follow-up, one participant died before the follow-up cognitive assessment; no participants were lost to follow-up or underwent kidney transplantation. The final analysis therefore included 130 participants, of whom 70 met the screening-defined incident cognitive impairment outcome and 60 did not.

Figure S5. Distribution of individual MoCA changes.

Observed change in MoCA score for all 130 participants, calculated as baseline minus 1-year MoCA. Participants are ordered by observed change; positive values indicate decline and negative values indicate improvement. Horizontal reference lines indicate no decline (0 points) and declines of 2 and 3 points. Colors indicate whether the participant met the primary screening-defined incident cognitive impairment outcome at follow-up.
